# Evaluation of Antimicrobial Activity of Novel Chimeric M-PEX12 Peptide Against *Acinetobacter baumannii*

**DOI:** 10.5812/ijpr-154484

**Published:** 2025-04-14

**Authors:** Yasin Rakhshani, Hamideh Mahmoodzadeh Hosseini, Seyed Ali Mirhosseini, Fatah Sotoodehnejadnematalahi, Jafar Amani

**Affiliations:** 1 Department of Biology, SR.C., Islamic Azad University, Tehran, Iran; 2 Applied Microbiology Research Center, Biomedicine Technologies Institute, Baqiyatallah University of Medical Sciences, Tehran, Iran

**Keywords:** Antimicrobial Peptides, *Acinetobacter baumannii*, Chimeric Peptide, Antibiotic Resistance

## Abstract

**Background:**

* Acinetobacter baumannii*-induced nosocomial pneumonia and its associated biofilm infections pose significant clinical challenges due to high rates of antibiotic resistance. Traditional antibiotic treatments encounter numerous obstacles, making antimicrobial peptides (AMPs) a promising alternative for controlling such pathogens. The emergence of multidrug-resistant strains necessitates the exploration of innovative therapeutic strategies.

**Objectives:**

We recently designed a novel hybrid peptide, M-PEX12, which exhibits antimicrobial activity and low toxicity in vitro. To confirm its therapeutic potential, we evaluated it in both in vitro and in vivo settings.

**Methods:**

M-PEX12 was evaluated using time-kill kinetics, thermal stability, reactive oxygen species (ROS) generation, biofilm inhibition assays, scanning electron microscopy (SEM), cytotoxicity tests, and virulence gene expression analysis. Its in vivo activity against *A. baumannii *was also assessed in an animal model.

**Results:**

The time-kill kinetics assay indicated that exposure to M-PEX12 at 1x minimum inhibitory concentration (MIC) (33/154) and 2x MIC resulted in over 95% reduction in bacterial populations within 30 minutes. Notably, the bacteria did not develop resistance to increased temperatures. M-PEX12 effectively disrupted biofilm formation at various concentrations. Field emission SEM revealed significant ultrastructural deformities in *A. baumannii *cell walls. Treatment with M-PEX12 increased production of intracellular ROS and decreased cell viability in a concentration-dependent manner. Cytotoxicity assays showed no significant effect on *HEK293* cell viability. Additionally, expression levels of *omp33*, *csuE*, *bfmR*, and *ompA* were significantly reduced. The antimicrobial efficacy of M-PEX12 was confirmed in vivo.

**Conclusions:**

M-PEX12 exhibited significant antimicrobial activity and low toxicity in a mouse model, suggesting its potential as a treatment for drug-resistant bacterial infections.

## 1. Background

The discovery and widespread use of antibiotics since the early 20th century have saved millions of lives worldwide. However, the development of novel antibiotics has slowed since the 1960s, with many new antibiotics being chemical variations of older, conventional compounds (1). One such concerning pathogen is *Acinetobacter baumannii*, a gram-negative opportunistic bacterium. *Acinetobacter baumannii *is closely monitored by health authorities due to its high incidence, particularly among immunocompromised populations. This pathogen is a leading cause of nosocomial (hospital-acquired) and community-acquired pneumonia, as well as skin and urinary tract infections, bacteremia, and surgical site infections (2). It has also demonstrated a significant increase in the prevalence of infections over recent decades. Despite having only a limited number of "traditional" virulence factors, the mechanisms underlying the success of this pathogen remain of great interest. There is an urgent need to find novel antimicrobial agents that control infections caused by multidrug-resistant (MDR) bacteria such as *A. baumannii*, *Staphylococcus aureus*, *Pseudomonas aeruginosa*, and *Klebsiella pneumoniae* (3).

Naturally occurring antimicrobial peptides (AMPs) produced by various tissues and cell types in organisms are considered promising sources for the development of new antimicrobial drugs. These AMPs represent an attractive class of molecules with the potential to combat pathogenic microorganisms (4). Most AMPs are oligopeptides consisting of 5 to 100 amino acids. They have a positive net charge, typically ranging from +2 to +11, and a significant proportion, typically around 50%, of hydrophobic residues (5). The AMPs are widespread in unicellular organisms, plants, and animals. More than 1,600 AMPs have been identified in a wide range of organisms. AMPs are active against a wide variety of microbes, including bacteria, fungi, unicellular protozoa, and viruses. The first identified AMP, gramicidin, was discovered in 1939 from the soil bacterium Bacillus brevis. It exhibited both in vitro and in vivo antibacterial activity against many gram-positive bacteria (6).

The AMPs target bacterial cell membranes, causing disintegration of the lipid bilayer, which leads to cytoplasmic leakage and bacterial death. Other reported mechanisms of antimicrobial action include the delocalization of membrane proteins, alteration of cytoplasmic membrane septum formation, and inhibition of cell wall, DNA, RNA, and protein synthesis (7). Although many potent AMPs have been identified, there are still major obstacles on the path to their clinical use. These obstacles include a lack of knowledge about their mechanisms of action, high-level toxicities of some AMPs, a lack of selectivity against specific strains, and high production costs (8). Therefore, the design of novel potent AMPs that exhibit low toxicities and in vivo susceptibility, as well as the identification of the mechanisms of AMP-membrane interactions, are important for the development of new antimicrobial agents.

We recently reported the design of a hybrid peptide AMP called M-PEX12, which was created by merging segments of two predefined peptides (MDC and Pexiganan) that were most similar in terms of amino acid type and order. The M-PEX12 peptide (amino acid sequence: GWLKFKKKVAILTDIIQAVALG) demonstrated promising performance, with a minimum inhibitory concentration (MIC) of 33.154 µM, a minimum bactericidal concentration (MBC) of 41.442 µM, and no hemolysis of human red blood cells. Additionally, results from circular dichroism (CD) analysis and molecular simulation (MD) were reported in a previous study.

## 2. Objectives

In the present study, we further evaluated the M-PEX12 peptide in vitro and in vivo to confirm the previously reported findings.

## 3. Methods

### 3.1. Time-Kill Kinetics

M-PEX12, synthesized by Genscrip in China with a purity of 97.8%, demonstrated effective antimicrobial activity against *A. baumannii *ATCC 19606. This bacterial strain was obtained from Baqiyatallah Hospital, with a MIC of 33.154 µM and a MBC of 41.442 µM. The bacteria were cultured in Muller-Hinton broth medium (MHB) at 37°C for 24 hours. Subsequently, the cultured bacteria were diluted to a turbidity equivalent to 0.5 McFarland and further diluted to achieve a bacterial concentration of 5 × 10^5^ CFU/mL per well (9).

To assess the dynamic effect of the antimicrobial agents at different time points, a killing kinetic assay was conducted. In this assay, *A. baumannii *at a concentration of 5 × 10^5^ CFU/mL in 96-well plates was mixed with 1/2x MIC, 1x MIC, and 2x MIC of the M-PEX12 peptide and incubated at 37°C for 0, 30, 60, 120, 240, and 360 minutes. Colony-forming units (CFU) were counted after 24 hours of incubation at 37°C. In this test, meropenem was used as a positive control, and a negative control was conducted without the peptide. This experiment was performed in triplicate (10).

### 3.2. Thermal Stability Assay

The thermal stability of the M-PEX12 peptide was evaluated by exposing it to various temperatures (4°C, 20°C, 40°C, 60°C, and 80°C) for 30 minutes. The experimental procedure involved adding 50 μL of the peptide and 50 μL of culture medium to microtubes, incubating them at the specified temperatures, and then adding 80 μL of culture medium and 20 μL of an *A. baumannii *suspension (5 × 10^5^ CFU/mL) to reach a final volume of 200 μL. The microtubes were then incubated overnight at 37°C, with a negative control (200 μL culture medium) and a positive control (180 μL culture medium + 20 μL bacteria). Antimicrobial activity was assessed using the agar diffusion method, where 5 μL of each sample was inoculated onto Mueller-Hinton agar (MHA) plates and incubated for 24 hours at 37°C to observe the presence of antimicrobial activity. The entire thermal stability analysis was performed in triplicate (11).

### 3.3. Reactive Oxygen Species Generation in Acinetobacter baumannii

To assess reactive oxygen species (ROS) levels in *A. baumannii *after treatment with the M-PEX12 peptide, a DCFH2-DA staining assay was conducted. *Acinetobacter baumannii *was cultured in MHB for 24 hours, followed by treatment with M-PEX12 at 1x and 2x MIC concentrations. Control samples included 1x phosphate-buffered saline (PBS) as a negative control and meropenem (100 μg/mL) as a positive control. After 2 and 4 hours of incubation at 37°C, samples were centrifuged, washed, and resuspended. They were divided into four tubes: One without dye, one with DCFH-DA dye, one with propidium iodide (PI) dye, and one with both. DCFH-DA tubes were incubated for 45 minutes at 37°C, followed by the addition of PBS and centrifugation. After resuspension, PI dye was added to the appropriate tubes, and flow cytometry was used to analyze ROS levels in the samples (12).

### 3.4. Ability of M-PEX12 Against Acinetobacter baumannii Biofilms Effects on Biofilm Formation

To evaluate the inhibitory effect of the M-PEX12 peptide on biofilm formation by *A. baumannii* ATCC 19606, a 96-well plate assay was conducted. Pre-grown *A. baumannii *was incubated with varying concentrations of the peptide (1/2x MIC, 1x MIC, 2x MIC, and 4x MIC) in tryptic soy broth (TSB) with 1% (w/v) sucrose, achieving a final bacterial concentration of 5 × 10^6^ CFU/mL. Negative control wells contained TSB with sucrose but no bacteria, while positive controls included TSB with sucrose and bacteria. After 24 hours of incubation at 37°C, the medium was discarded, and the wells were washed with PBS. Biofilms were fixed with methanol, air-dried, and stained with 0.1% crystal violet. Following a rinse with water, 200 μL of 95% ethanol was added to each well to solubilize the dye. The absorbance was measured at 595 nm using a Bio-Rad Microplate Reader to quantify biofilm biomass, with the assay performed in triplicate (13).

### 3.5. Scanning Electron Microscope Observations

After incubating *A. baumannii *at 37°C for 24 hours, the bacteria were diluted to a concentration of 5 × 10^6^ CFU/mL and treated with 2x and 4x MIC concentrations of the M-PEX12 peptide, with meropenem as a positive control and PBS as a negative control. Following a 9-hour incubation at 25°C, the samples were centrifuged, and the bacterial pellets were washed with PBS and spread onto slides. After drying, the slides were fixed in 4% formaldehyde for 2 hours, then prefixed in 8% formaldehyde overnight. The slides were washed, dehydrated through a series of alcohol concentrations (50% to 100%), and coated with platinum to prevent charging during imaging. Secondary electron images were captured using a Zeiss Supra 55VP microscope at low electron energies between 2 keV and 2.5 keV (14).

### 3.6. Determination of Peptides Toxicity on HEK293 Cell Line by MTT Assay

To assess the toxicity of the M-PEX12 peptide, *HEK293* cells (obtained from the Pasteur Institute of Iran) were cultured at 8,000 cells per well in 96-well plates for 24 hours in Dulbecco's Modified Eagle Medium (DMEM) with 10% fetal bovine serum and antibiotics. The cells were treated with varying concentrations of M-PEX12 (1/2 MIC, MIC, 2 MIC) or left untreated as a control. After incubation, cells were washed with PBS, and MTT solution was added to each well for a 4-hour incubation at 37°C. Following this, the supernatant was removed, dimethyl sulfoxide was added to dissolve the formazan crystals, and absorbance was measured at 570 nm using a BioTek Synergy^TM^ 2 microplate reader. Results were reported as the average of at least three experiments, with control cell viability set at 100% (15).

### 3.7. Real-time PCR

#### 3.7.1. RNA Extraction and Complementary DNA Synthesis and In Vitro Virulence Gene Expression

*Acinetobacter baumannii* was treated with the M-PEX12 peptide at half the minimum inhibitory concentration (1/2 MIC) and incubated at 37°C for 24 hours. Total mRNA was isolated using the TRIzol method, followed by DNase I treatment to remove any DNA. The RevertAid First Strand cDNA Synthesis Kit was used to generate complementary DNA (cDNA) libraries from 1 μg of total RNA, which were then utilized for quantitative real-time reverse transcription PCR (qRT-PCR) assays (16).

#### 3.7.2. Reverse Transcriptase and Quantitative-PCR Assays

The expression of genes *ompA*, *omp33*-36, *csuE*, *bmfR*, and the housekeeping gene DNA gyrA was assessed using the RT-PCR assay. The cDNA samples were analyzed using the qRT-PCR assay with specific primer sets as listed in Table 1. The Qiagen real-time PCR system (BIOER Technology Co., China) was utilized to perform the qRT-PCR. The qRT-PCR assay was performed under the following conditions: An initial denaturation step at 95°C for 3 minutes, followed by 40 cycles of denaturation at 95°C for 15 seconds, annealing at 60°C for 15 seconds, and extension at 72°C for 30 seconds. The reaction mixture contained 10 µL of SYBR^®^ premix Ex Taq^TM^ II (Tli RNaseH Plus) (TaKaRa Bio Inc., Japan), 2 µL of gene-specific forward and reverse primers (2.5 pmol each), cDNA template (500 ng), and 7 µL of ddH_2_O, making a total volume of 20 µL. The expression of the target virulence genes was normalized using *DNA gyrase A* as an internal control (17). The expression of the target genes, after normalization with housekeeping genes, was log2 transformed prior to analysis. The data were then subjected to a one-way analysis of variance (ANOVA) using the linear 2^-∆∆CT^ dataset. The least significant difference (LSD) method was employed to examine the differences between outcome groups.

**Table 1.** Characteristics of Sequence Primers

Primer Names	Sequences (5'- 3')	Amplicon Size (bp)	Gene ID	Ref.
**omp A**		182	KF195966.1	(18)
Forward	ATTTACCAGGATGGGCCGTG			
Reverse	GCGCCACAACCAAGCAATTA			
**omp 33-36**		194	AJ831523.2	(19)
Forward	GTTTTCTTGACCGAATGCACC			
Reverse	GTTTTCTTGACCGAATGCACC			
**csu E**		103	A0AAP1W7J4	(20)
Forward	TTGTGGGAATCGGGGTGTTCTTTG			
Reverse	GAGAGTGAACCAAGCGAGTCTGC			
***bfmR***		200	66398311	(21)
Forward	CTGGTAGGTAATGCAGTTCG			
Reverse	GAGAGACCCAAACCATAACC			
***DNA ** **gyras** ** A***		122	CT025946.2	(22)
Forward	AAGGCCGTCCAATCGTGAA			
Reverse	AACCGTACCAGAAGCTGTC			

### 3.8. Animal Study Design

#### 3.8.1. Animals

Male adult BALB/c mice (8-week-old), weighing 20 - 25 g, were purchased from the Baqiyatallah University of Medical Science, Tehran, Iran, and approved by the Animal Studies Committee of the mentioned institution. All animals were housed in polypropylene cages in a temperature-controlled room (24 ± 2°C) with 30 - 35% relative humidity and a 12-hour light/dark cycle. All procedures involving animals were in accordance with the national guidelines for the care and use of laboratory animals in scientific affairs provided by the Iranian Ministry of Health and Medical Education (2021). The guideline complies with the ARRIVE (animal research: Reporting of in vivo experiments) guidelines (permit code: IR.IAU.SRB.REC.1399.117) (23).

#### 3.8.2. Establishment of the Peritonitis Infection Model and Determination of Effective Dose

The peritonitis mouse model was created by administering an overnight cultured suspension of *A. baumannii *(ATCC 19606) intraperitoneally. Mice were divided into five groups, each receiving different bacterial doses (1 × 10^4^ to 1 × 10^9^ CFU/mL) in 500 µL volumes, with three mice per group. Blood samples were collected from the mice's eyes every 24 hours for 48 hours, and 20 µL of blood was cultured on MHA. The lowest bacterial concentration that induced septicemia without causing mortality was identified for further investigation of the antimicrobial effects of the peptide used (24).

#### 3.8.3. In Vivo Efficacy of M-PEX12 Against Acinetobacter baumannii Peritonitis Mouse Model

The in vivo efficacy of M-PEX12 was assessed in a peritoneal infection model using BALB/c mice infected with a bacterial suspension of *A. baumannii *at 1 × 10^8^ CFU/mL. Mice were divided into four groups of six, with the bacteria centrifuged, washed, and resuspended in PBS before intraperitoneal (i.p.) injection. After one hour, M-PEX12 was administered at two doses (1 MBC/kg and 2 MBC/kg) to the first two groups, while the third group received a standard dose of meropenem as a positive control, and the fourth group was treated with PBS. Blood samples were collected every 24 hours for 48 hours, cultured on MHA, and bacterial colonies were counted. After 72 hours, all mice were euthanized, and tissue lavages were homogenized and plated on MHA for CFU evaluation (16, 25).

### 3.9. Statistical Analysis

Statistical analysis was conducted using SPSS software version 22 (SPSS Inc., Chicago, IL, USA). Differences were deemed significant when the P-value was less than 0.05 (P < 0.05). The Mann-Whitney U test was utilized for statistical analysis. This non-parametric test was chosen for its suitability in analyzing data that do not conform to the assumptions of normality. Additionally, non-parametric tests are less susceptible to outliers, thus enhancing result reliability. In addition to the one-way ANOVA, other tests were employed for analysis. One-way ANOVA was specifically chosen due to the involvement of variables with three categories. One-way ANOVA was used for testing time-kill kinetics, and the Mann-Whitney U test was used for pairwise c*ompA*risons.

## 4. Results

### 4.1. Time-Kill Kinetics

**Figure 1.**
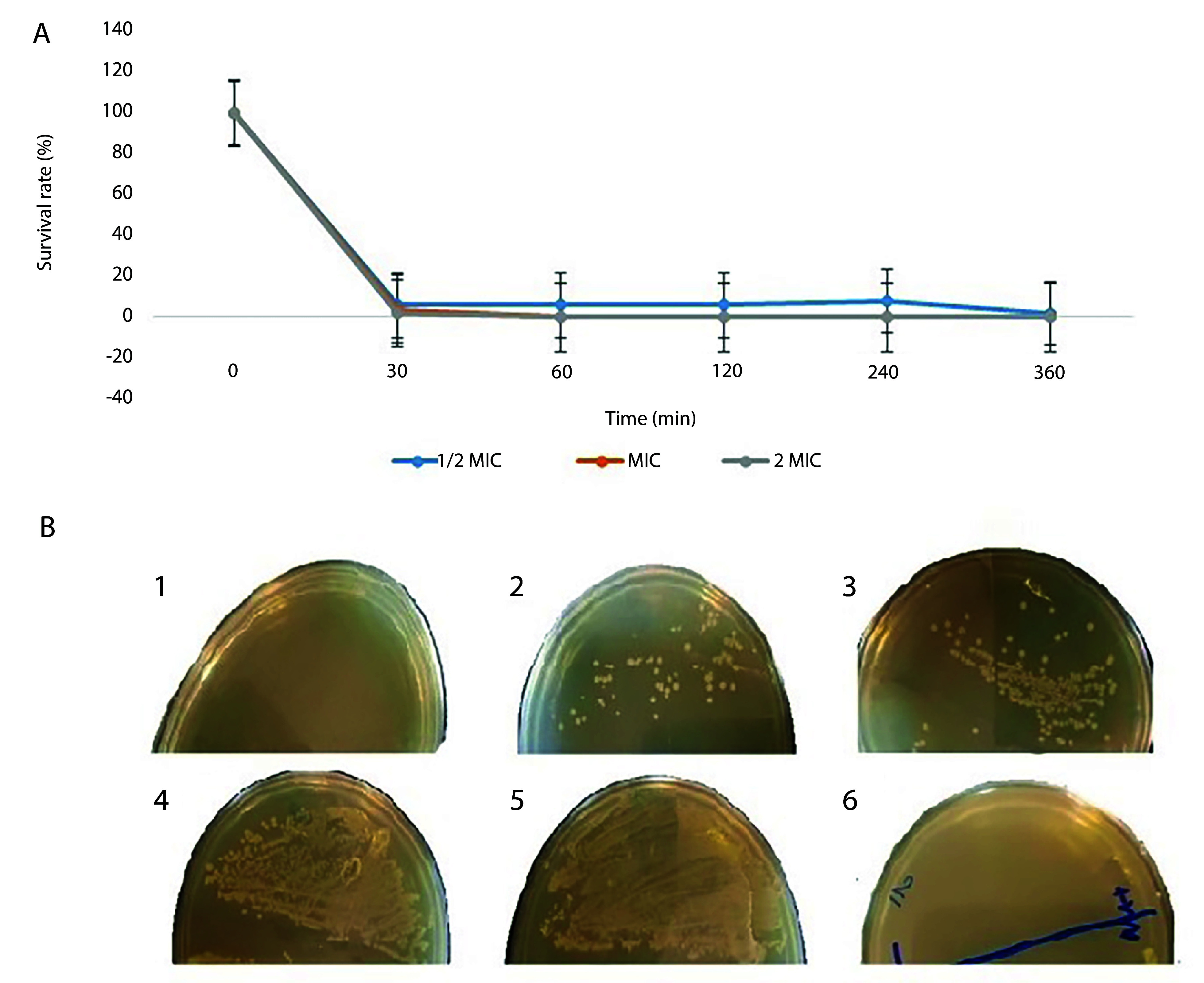
A, The survival percentage of *Acinetobacter baumannii *bacteria after treatment with the M-PEX12 peptide was measured at different time points. Significant differences in survival rates were observed between groups at the following time points: Thirthy minutes (P < 0.001), 60 minutes (P < 0.001), 120 minutes (P = 0.005), 240 minutes (P < 0.001), and 360 minutes (P = 0.001); B, the growth of *A. baumannii *bacteria was assessed at different temperatures (1, 2, 3, 4, 5) from left to right, corresponding to temperatures of 4, 20, 40, 60, and 80 degrees Celsius. 6, A negative control was also included.

According to the results of the time-kill test, the M-PEX12 peptide was able to significantly reduce the number of live *A. baumannii *bacteria within 1 hour after treatment at a concentration of 1/2 MIC. Additionally, this peptide demonstrated a reduction of the bacterial population by more than 95% after 30 minutes of exposure to 1x MIC and 2x MIC of M-PEX12 peptide. The statistical analysis revealed significant differences in survival rates across the peptide concentration groups at multiple time points. Specifically, the between-group c*ompA*rison showed significant differences at 30 minutes (P < 0.001), 60 minutes (P < 0.001), 120 minutes (P = 0.005), 240 minutes (P < 0.001), and 360 minutes (P = 0.001). These results indicate that the varying peptide concentrations had a statistically significant impact on the survival rates c*ompA*red to the control group (Figure 1A). 

### 4.2. Thermal Stability Assay

Although most AMPs are stable against high temperatures, the results indicated that the peptide M-PEX12 did not show resistance to high temperature (Figure 1B). 

### 4.3. Reactive Oxygen Species Generation in Acinetobacter baumannii Upon Exposure to M-PEX12

**Figure 2.**
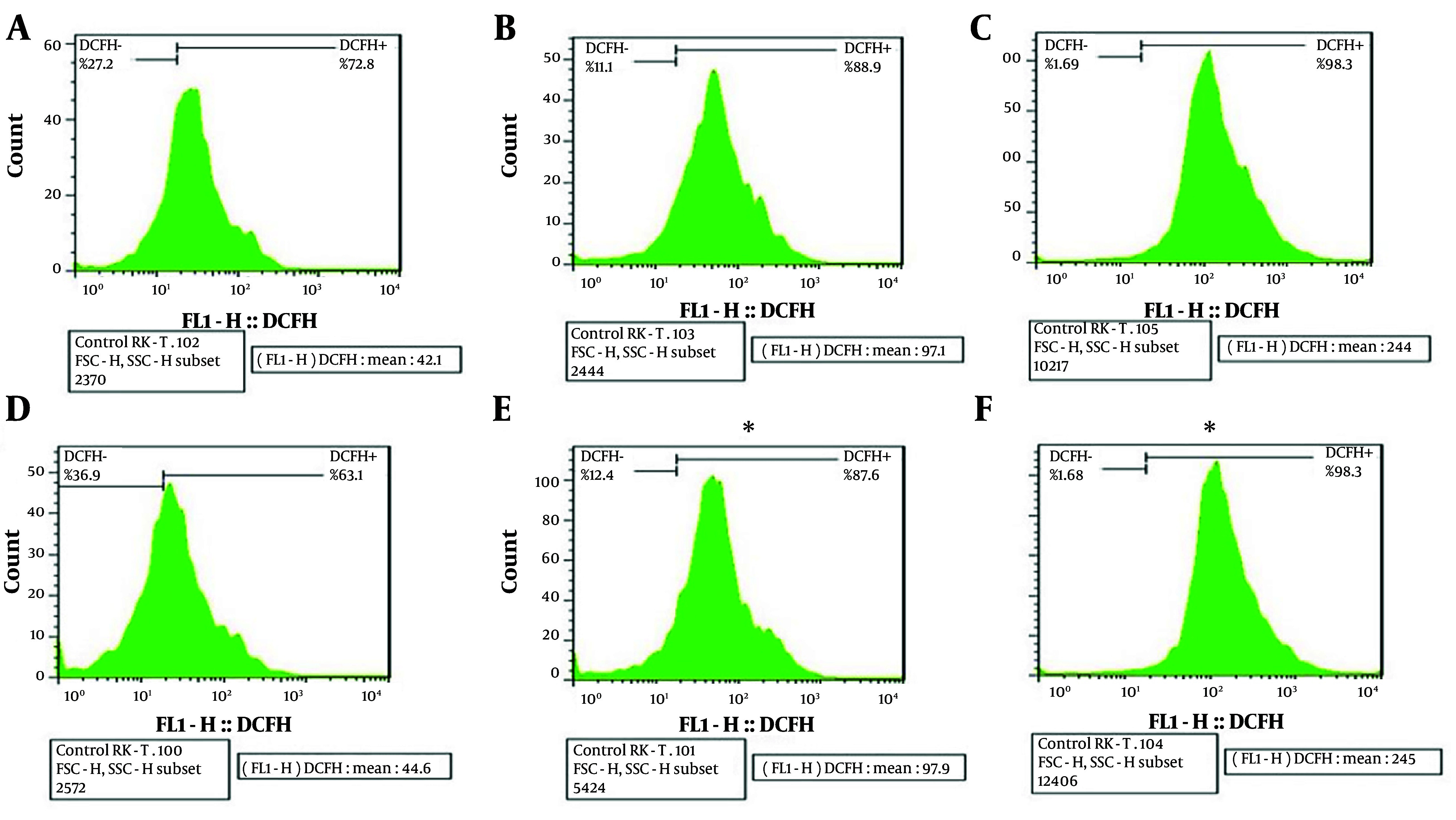
The expression level of DCFH dye is indicated on the right side of the graph in the upper section, and beneath each graph, a numerical value reported as mean fluorescence intensity (MFI) represents the intensity of color in each sample for c*ompA*rison across multiple graphs. In the above graph, A, B, and C, respectively represents the negative control, minimum inhibitory concentration (MIC) peptide concentration, and 2xMIC peptide concentration at 2 hours of bacterial and peptide treatment; similarly, D, E and F, respectively represents the negative control, MIC peptide concentration and 2x MIC peptide concentration at 4 hours of treatment (* P < 0.05).

The ROS production assay was conducted to assess the amount of ROS generated after exposing M-PEX12 to *A. baumannii *bacteria. The results showed that an increase in M-PEX12 concentration led to ROS generation in *A. baumannii*, resulting in bacterial cell death. The mean fluorescence intensity (MFI) value reported in each sample indicates the color intensity. In the negative control sample (bacteria without peptide treatment), the MFI was lower c*ompA*red to the treatment of bacteria with the peptide at the MIC and 2x MIC concentrations. This suggests that the peptide treatment had an impact on ROS generation. In the negative control sample, the MFI was 44.6. However, when the bacteria were treated with the peptide at MIC and 2x MIC concentrations, the MFIs were 97.1 and 244 after 2 hours, respectively. After 4 hours, the MFI in the negative control sample remained at 44.6, while the MFIs for the MIC and 2x MIC peptide treatments were 97.6 and 245, respectively. These increasing MFI values indicate a higher generation of ROS. Consequently, as the peptide concentration increased in the presence of bacteria, the rate of bacterial death also increased. The results indicated that the P-value was less than 0.05 (P < 0.05), suggesting a statistically significant difference in ROS production between the different concentrations and the control group. This observation is depicted in Figure 2. 

### 4.4. Inhibitory Effect of M-PEX12 on Acinetobacter baumannii Biofilm Formation

**Figure 3.**
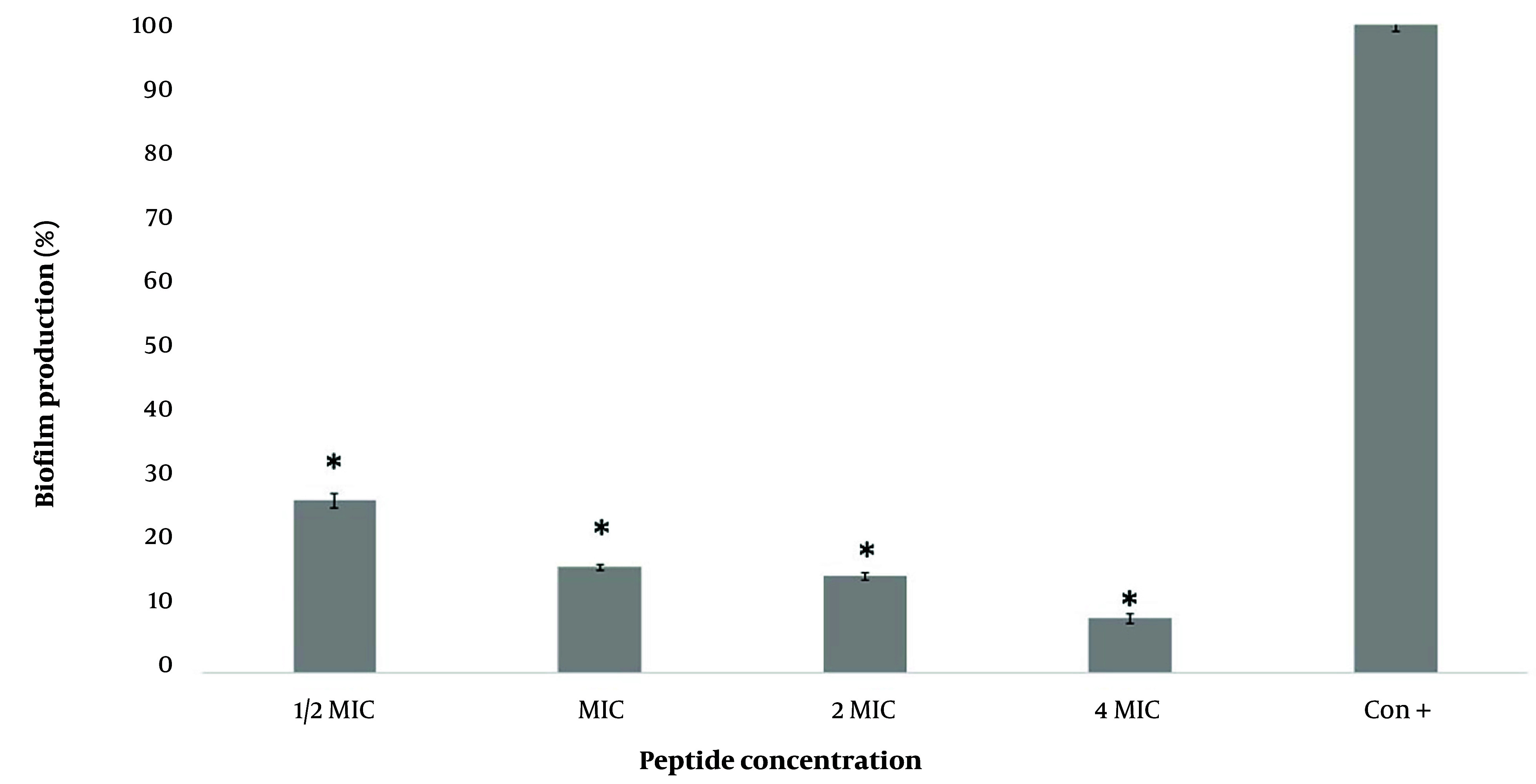
Biofilm formation by *Acinetobacter baumannii *bacteria at different concentrations of M-PEX12 (* P < 0.05)

Figure 3 demonstrates the concentration-dependent inhibitory effect of M-PEX12 on biofilm formation by *A. baumannii *ATCC 19606. Treatment with 1/2x MIC, 1x MIC, 2x MIC, and 4x MIC concentrations of M-PEX12 for 24 hours resulted in biofilm production rates of 26.5%, 16.2%, 14.8%, and 8.3%, respectively. These findings highlight a reduction in biofilm formation and demonstrate the effectiveness of M-PEX12 in inhibiting biofilm formation by *A. baumannii*. Based on the statistical analysis, biofilm production in the presence of peptides shows a significant reduction c*ompA*red to the absence of peptides (negative control) (P < 0.05).

### 4.5. Scanning Electron Microscope Observations

#### 4.5.1. Effects of M-PEX12

**Figure 4.**
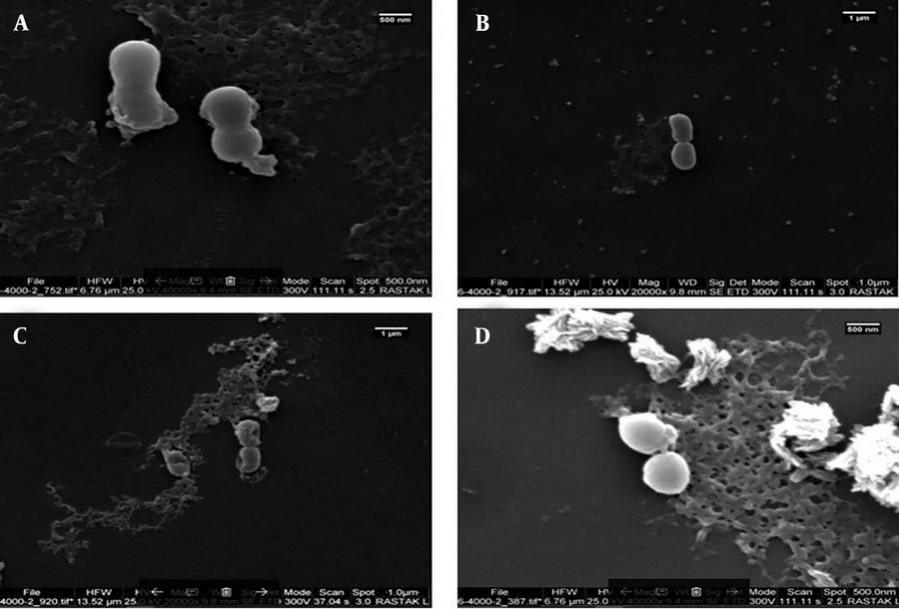
Scanning electron microscopy (SEM) images before and after peptide treatment. A and B, Show *Acinetobacter baumannii *bacteria before peptide treatment at different magnifications and time points; C, shows *A. baumannii *after a 2-hour treatment with the peptide at a concentration of 2x minimum inhibitory concentration (MIC); D, shows *A. baumannii *after a 4-hour treatment with the peptide at a concentration of 4x MIC (25)

The change in bacterial morphology after treatment with peptides was directly observed by scanning electron microscopy (SEM) (Figure 4). The untreated *A. baumannii* ATCC 19606 bacteria exhibited a bright and smooth surface. After treatment with 2x MIC and 4x MIC M-PEX12, the membrane surface of the *A. baumannii *cells became completely roughened and corrugated, and even induced atrophy and fracture. Deformed cell morphology and leakage of cellular contents demonstrated the effects of these peptides on *A. baumannii*.

### 4.6. Determination of Peptides Toxicity on HEK293 Cell Line by MTT Assay

**Figure 5.**
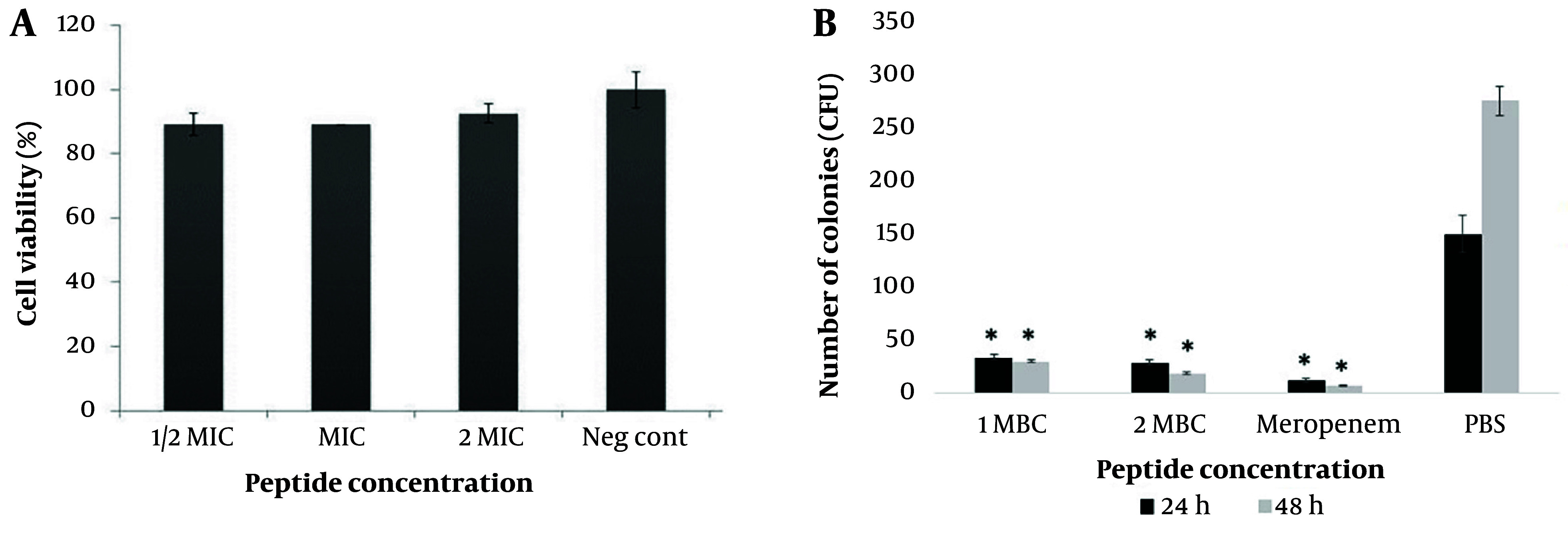
A, In the investigation on the cytotoxic effect of the M-PEX12 peptide on the *HEK293* cell line (P > 0.05); B. investigation of the antimicrobial activity of the novel chimeric peptide M-PEX12 in a mouse model of sepsis caused by *Acinetobacter baumannii *at 24 and 48 hours post-infection (* P < 0.05).

In this study, the cytotoxicity of M-PEX12 on *HEK293* cells was investigated at different concentrations. Based on the toxicity/concentration chart (Figure 5A), no significant difference in cell viability was observed c*ompA*red to the control sample at any concentration (P > 0.05). This finding indicates the low toxicity of M-PEX12 at different concentrations (1/2x MIC, 1x MIC, 2x MIC) on this particular cell line.

### 4.7. Analysis of Differential Gene Expression

To analyze the relative expression of virulence genes, *A. baumannii *was treated with 1/2 MIC M-PEX12 peptide. The statistical results indicated that the relative expression levels of *omp33* - 36, *csuE*, bmf, and *ompA* genes were significantly decreased, with fold changes of 0.044, 0.35, 0.7, and 0.7, respectively, c*ompA*red to the negative control (P < 0.05).

### 4.8. In Vivo Efficacy of M-PEX12 Against Acinetobacter baumannii Peritonitis Mouse Model

The in vivo antimicrobial activity of M-PEX12 was evaluated using an *A. baumannii *mouse model. Various concentrations of bacteria were administered to the mice, and it was observed that septicemia was induced at a concentration of 1 × 10^8^ CFU/mL. Lower concentrations (1.0 × 10^4^ CFU/mL, 1.0 × 10^6^ CFU/mL, and 1.0 × 10^7^ CFU/mL) did not cause septicemia. Therefore, the concentration of 1 × 10^8^ CFU/mL was chosen to investigate the antimicrobial efficacy of M-PEX12 in the mouse model. The results showed that M-PEX12 at 1x MIC (33.154 mM) and 2x MIC (66.3 mM) doses effectively reduced *A. baumannii *bacteria in vivo. Within 48 hours, the peptide significantly decreased the colony-forming unit (CFU) count in the infected mice. After 72 hours, no bacterial load was detected in the peritoneal lavage solution (Figure 5B). The statistical analysis revealed that the differences in CFU counts between the treated and control groups were significant (P < 0.05), supporting the effectiveness of M-PEX12 as an antimicrobial agent against *A. baumannii *in vivo.

## 5. Discussion

*Acinetobacter baumannii* is a significant public health threat due to its virulence and ability to cause epidemics. Its capacity to form biofilms and survive in harsh environmental conditions poses challenges in healthcare settings, facilitating the spread and severity of *A. baumannii *infections (26, 27). The AMPs are versatile immune molecules that can serve as potential alternatives to conventional antibiotics, though their natural forms are limited by susceptibility to proteolytic degradation (28). To address the limitations of natural AMPs, researchers have focused on designing and synthesizing shorter, optimized peptide sequences that exhibit enhanced antimicrobial activity and reduced toxicity, a promising approach for discovering and developing effective antimicrobial agents (29).

The present study further evaluated the in vitro and in vivo efficacy of the peptide M-PEX12, which demonstrated a greater than 95% reduction in the *A. baumannii *population when exposed for 30 minutes at concentrations corresponding to 1x and 2x MIC. The rapid killing kinetics of M-PEX12 against *A. baumannii*, c*ompA*rable to other AMPs, may be attributed to its dual mechanisms of action involving both membrane disruption and interaction with intracellular targets. In a study by Prada-Prada et al., the newly designed AMP Ib-M exhibited a greater than 95% reduction in the *E. coli* O157:H7 population after a 4-hour exposure at 1x MIC (30).

This study found that the antibacterial agent M-PEX12 can potentially induce the excessive generation of ROS, including hydrogen peroxide (H_2_O_2_), hydroxyl radical (OH^-^), singlet oxygen (O_2_), and superoxide anion (O_2_^–^), in *A. baumannii *bacteria (31). The increased levels of ROS can damage bacterial proteins, lipids, and nucleotides, leading to programmed cell death. Although bacteria have some tolerance to ROS due to antioxidative stress response genes (32), M-PEX12 can induce a lethal level of oxidative stress that overwhelms the bacterial defense mechanisms, ultimately resulting in the death of the *A. baumannii *cells.

Taken together, the M-PEX12 AMP exhibits effective anti-biofilm activity against *A. baumannii *ATCC 19606 through various mechanisms, including disrupting bacterial membranes, interfering with cell signaling, and degrading the extracellular matrix, resulting in a dose-dependent inhibition of biofilm formation (33). Molecular dynamics simulations (previous study) and SEM analysis revealed that M-PEX12, with its physicochemical properties of short chain, α-helix, net positive charge, and amphipathic nature, disrupts bacterial membranes and alters the surface morphology of *A. baumannii*, leading to pore formation and significant damage, without exhibiting toxicity against host cells. In a study by Wang et al., synthetic peptides derived from butterflies exhibited antimicrobial effects by causing significant changes in the structure and properties of microbial cell membranes and nuclei, suggesting their ability to penetrate cell membranes and interact with nucleic acids (34).

In vitro assessments showed that the AMP M-PEX12 exhibited a favorable cytotoxicity profile, maintaining high viability of *HEK293* cells even at concentrations exceeding its minimum inhibitory concentration (MIC and twice the MIC of M-PEX12), suggesting its potential as a promising antimicrobial agent. The hemolysis assay results showed that M-PEX12 exhibited minimal hemolytic activity at the MIC concentration (0.72%) and a slightly higher level at 2x MIC (1%), supporting the safety profile of M-PEX12 and its potential for in vivo evaluation of efficacy against *A. baumannii *infection in a mouse model.

The study found that treating *A. baumannii *with the M-PEX12 peptide led to a significant decrease in the expression of virulence genes, likely due to the peptide's ability to penetrate bacterial cells and interact with nucleic acids and intracellular proteins, consistent with previous research by Liu et al. on the impact of AMPs on virulence gene expression (20). The study found that while the AMP M-PEX12 and the antibiotic meropenem both showed significant reductions in *A. baumannii *bacterial counts and improved survival in a mouse model, meropenem was more effective, suggesting further investigation is needed to enhance the efficiency of M-PEX12, potentially by optimizing the modifications made to the parent peptide sequence during the hybridization process.

The study by Nagarajan et al. found that unlike the antibiotic colistin, the AMP Ω76 does not possess nephrotoxic properties, suggesting Ω76 has potential to be used as an alternative or adjunctive therapy to colistin in clinical settings (25). The study by Jung et al. found that the AMPs SMAP-29, TP4, and its derivatives dN4 and dC4 exhibited prophylactic and therapeutic effects against *A. baumannii*-induced pneumonia, including the ability to inhibit and eliminate *A. baumannii *biofilms, suggesting their potential as treatment strategies (35). These findings validate the outcomes of the in vitro investigations and provide evidence that M-PEX12 holds significant promise for being formulated into an effective antimicrobial therapeutic agent.

Based on the statistical results of the conducted tests and the significant differences observed c*ompA*red to the control group (P < 0.05), the M-PEX12 peptide has the potential to effectively control the target bacteria. While the results with M-PEX12 are promising, several limitations must be acknowledged. The small sample size in our in vivo experiments may restrict the generalizability of our findings. Additionally, the specific strains of *A. baumannii *used may not fully represent the diversity encountered in clinical settings. Although our in vitro results are encouraging, the in vivo environment poses additional challenges that could affect M-PEX12's efficacy. The selection of strains and testing conditions may introduce biases, even though we aimed to minimize this by using well-characterized strains. Lastly, variations in environmental factors, such as temperature and pH, could influence outcomes despite our efforts to standardize these conditions.

In summary, our study introduces a novel approach to the design and prediction of AMPs. The peptides generated using our method demonstrated effective antibacterial activity in both laboratory experiments (in vitro) and living organisms (in vivo). However, it is important to acknowledge that synthetic peptides may not exhibit identical behavior in computational simulations (in silico) and laboratory investigations, as emphasized by our findings. Despite M-PEX12 exhibiting lower efficacy c*ompA*red to certain antibiotics against *A. baumannii*, it holds promise as an alternative treatment for *A. baumannii *infections, particularly considering the high prevalence of antibiotic resistance. These findings have significant implications for future research in the development and application of innovative antimicrobial therapies.

## Data Availability

The data presented in this study are uploaded during submission as a supplementary file and are openly available for readers upon request.
